# Measurement of HbA_1c_ and HbA_2_ by Capillarys 2 Flex Piercing HbA_1c_ programme for simultaneous management of diabetes and screening for thalassemia

**DOI:** 10.11613/BM.2017.030704

**Published:** 2017-08-28

**Authors:** Peifeng Ke, Jiawei Liu, Yan Chao, Xiaobin Wu, Yujuan Xiong, Li Lin, Zemin Wan, Xinzhong Wu, Jianhua Xu, Junhua Zhuang, Xianzhang Huang

**Affiliations:** 1 Department of Laboratory Science, Second Affiliated Hospital of Guangzhou University of Chinese Medicine, Guangzhou, China; 2 Second Clinical Medical College, Guangzhou University of Chinese Medicine, Guangzhou, China

**Keywords:** haemoglobin A_1c_, thalassemia, capillary electrophoresis (CE), haemoglobin A_2_

## Abstract

**Introduction:**

Thalassemia could interfere with some assays for haemoglobin A_1c_ (HbA_1c_) measurement, therefore, it is useful to be able to screen for thalassemia while measuring HbA_1c_. We used Capillarys 2 Flex Piercing (Capillarys 2FP) HbA_1c_ programme to simultaneously measure HbA_1c_ and screen for thalassemia.

**Materials and methods:**

Samples from 498 normal controls and 175 thalassemia patients were analysed by Capillarys 2FP HbA_1c_ programme (Sebia, France). For method comparison, HbA_1c_ was quantified by Premier Hb9210 (Trinity Biotech, Ireland) in 98 thalassaemia patients samples. For verification, HbA_1c_ from eight thalassaemia patients was confirmed by IFCC reference method.

**Results:**

Among 98 thalassaemia samples, Capillarys 2FP did not provide an HbA_1c_ result in three samples with HbH due to the overlapping of HbBart’s with HbA_1c_ fraction; for the remaining 95 thalassaemia samples, Bland-Altman plot showed 0.00 ± 0.35% absolute bias between two systems, and a significant positive bias above 7% was observed only in two HbH samples. The HbA_1c_ values obtained by Capillarys 2FP were consistent with the IFCC targets (relative bias below ± 6%) in all of the eight samples tested by both methods. For screening samples with alpha (α-) thalassaemia silent/trait or beta (β-) thalassemia trait, the optimal HbA_2_ cut-off values were ≤ 2.2% and > 2.8%, respectively.

**Conclusions:**

Our results demonstrated the Capillarys 2FP HbA_1c_ system could report an accurate HbA_1c_ value in thalassemia silent/trait, and HbA_2_ value (≤ 2.2% for α-thalassaemia silent/trait and > 2.8% for β-thalassemia trait) and abnormal bands (HbH and/or HbBart’s for HbH disease, HbF for β-thalassemia) may provide valuable information for screening.

## Introduction

Haemoglobin A_1c_ (HbA_1c_), the major form of all glycated haemoglobin species, is produced by the non-enzymatic addition of glucose residues to valine moieties at the N-terminal end of the haemoglobin (Hb) β-chain (*1*). HbA_1c_ concentrations are used as an index for long-term glycaemic control, and have recently been recommended for the diagnosis of diabetes mellitus (*2*-*4*). Various methods have been developed to measure HbA_1c_, however, their accuracy can be adversely affected by the presence of haemoglobinopathies (*5*, *6*). In addition, HbA_1c_ provides an estimate of blood glucose concentrations over a normal erythrocyte lifespan, and any conditions altering erythrocyte survival (*e.g.*, haemolytic anaemia, iron deficiency anaemia or haemoglobinopathies) may lead to misinterpretation of the HbA_1c_ result and hence misdiagnosis and mistreatment (*7*).

Thalassemia is the most common monogenetic disease caused by defects in the synthesis of one or more of the haemoglobin chains (*8*, *9*). Alpha (α-) thalassemia is caused by reduced or absent synthesis of α globin chains, including four main forms: α-thalassemia silent, α-thalassemia trait (minor), HbH (intermedia) and HbBart’s hydrops fetalis syndrome (major). Beta (β-) thalassemia is caused by reduced or absent synthesis of β globin chains, including three main forms: β-thalassemia trait (minor), β-thalassemia intermedia and β-thalassemia major. Several studies have suggested that HbA_1c_ measurement may be affected by thalassemia, which is probably due to different methods used, and the genotypes of samples (*10*-*16*). In addition, the imbalance of globin chains in thalassemia can lead to haemolysis and impaired erythropoiesis. A decrease of erythrocyte lifespan has been reported in β-thalassemia (*17*). Therefore, it is useful to be able to screen for thalassemia while measuring HbA_1c_. Detection of thalassaemia is especially important for the identification of couples at risk of having a child with thalassemia major.

Recently, Sebia (Evry Cedex, France) introduced a capillary electrophoresis (CE) method for the determination of HbA_1c_ using Capillarys 2 Flex Piercing (Capillarys 2FP). Our previous study indicated that HbA_1c_ concentrations measured by Capillarys 2FP system in normal samples, without haemoglobinopathies, showed high correlation and concordance with those obtained using a boronate affinity high-performance liquid chromatography (HPLC) system, *i.e.* Premier Hb9210 (Trinity Biotech Plc, Bray, Ireland) (*5*). Further accuracy verification demonstrated a great consistency with International Federation of Clinical Chemistry and Laboratory Medicine (IFCC) reference method (HPLC/CE) values (*5*). However, the effect of thalassemia on the CE method is unclear. The HbA_1c_ programme on Capillarys 2FP could provide a rapid and reliable separation of HbA_2_, and HbA_2_ results are systematically lower compared to those obtained with the haemoglobin programme, with an average bias of 0.29% (*10*). HbA_2_ plays an important role in screening programme for thalassemia: its decrease might reveal α-thalassemia while its increase might indicate β-thalassemia (*18*). Therefore, in this study, we evaluated the effect of thalassemia on HbA_1c_ measurement using Capillarys 2FP by comparison with the Premier Hb9210 and IFCC reference methods, and, furthermore, assessed the HbA_1c_ programme on Capillarys 2FP for thalassemia screening.

## Materials and methods

### Subjects

The Research and Ethics committees of our institution approved this study. All subjects were informed on the study contents and provided written consents for participation. Blood samples were obtained from the Clinical Laboratory of the Second Affiliated Hospital of Guangzhou University of Chinese Medicine between June 2014 and August 2014. These samples included 498 healthy adults (normal controls) and 175 patients with thalassemia. The 175 thalassemia patients included 51 with α-thalassemia silent, 57 with α-thalassemia trait, 7 with HbH and 60 with β-thalassemia trait. Whole blood samples were collected in EDTA-containing tubes (2.0 mL, BD Franklin Lakes NJ, USA), divided into small aliquots and stored at - 80°C before analysis as previously described (*19*, *20*).

Normal control samples were selected from subjects undergoing routine laboratory check-up examinations. Complete blood count (CBC) parameters were measured with BC-6800 (Mindray Medical Electronics Co., Shenzhen, China). Inclusion criteria were as follows: Hb concentrations > 130 g/L (in men) or > 115 g/L (in women), mean corpuscular volume (MCV) > 80 fL, HbF < 5% without Hb disorders (Hb phenotype analysis was performed by Bio-Rad Variant II system (Bio-Rad, Japan) using the beta thalassemia programme). Thalassemia samples were collected from routine laboratory test samples for thalassemia. Gap-polymerase chain reaction (PCR) was used to identify the α-thalassemia deletion mutations, and PCR reverse-blot hybridization was used to detect β-thalassemia point mutations (YANENG Bioscience Co. Ltd., Shenzhen, China) (*21*, *22*).

### Method comparison

The HbA_1c_ values of 98 thalassemia patients (29 α-thalassemia silent, 41 α-thalassemia traits, 7 HbH and 21 β-thalassemia traits), were quantified by Capillarys 2FP and Premier Hb9210 systems according to the manufacturers’ instructions. The analysers were calibrated once prior to any sample analysis.

Because glycated and non-glycated haemoglobins are separated regardless of haemoglobin species, thalassemia is unlikely to interfere with the IFCC reference method and boronate affinity chromatography method (*23*). The boronate affinity HPLC method Premier Hb9210 was used as the comparative method. For verification, eight thalassemia samples were sent to Shanghai Center for Clinical Laboratory and confirmed by the IFCC reference method HPLC/CE.

### Thalassemia screening

A total of 498 normal controls and 168 thalassemia samples (51 α-thalassemia silent, 57 α-thalassemia traits, and 60 β-thalassemia traits) were analysed by Capillarys 2FP HbA_1c_ programme.

### Statistical analysis

For method comparison, the differences between two methods (Capillarys 2FP vs Premier Hb9210) were presented in a Bland-Altman plot. The relative bias was calculated by the Capillarys 2FP value against the comparative method (Premier Hb9210) value, and > ±7% was considered clinically significant (National Glycohemoglobin Standardization Programme (NGSP) criterion) (*24*). For verification testing, the relative bias was calculated by the Capillarys 2FP value against the IFCC reference method value, and the proficiency testing acceptance limit of ± 6% provided by the College of American Pathologists (CAP) was set as the accuracy limit (*25*).

Measurement data of HbA_2_ were normally distributed and the independent sample t-test was performed to detect statistical difference between any two groups. Receiver operating characteristic curve (ROC) was applied to determine the best cut-off for screening α- and β-thalassemia. A P value of < 0.05 was considered statistically significant. Statistical analysis was performed using the MedCalc version 14.8.1 (MedCalc Software, Ostend, Belgium).

## Results

### Method comparison

**Figure 1:**
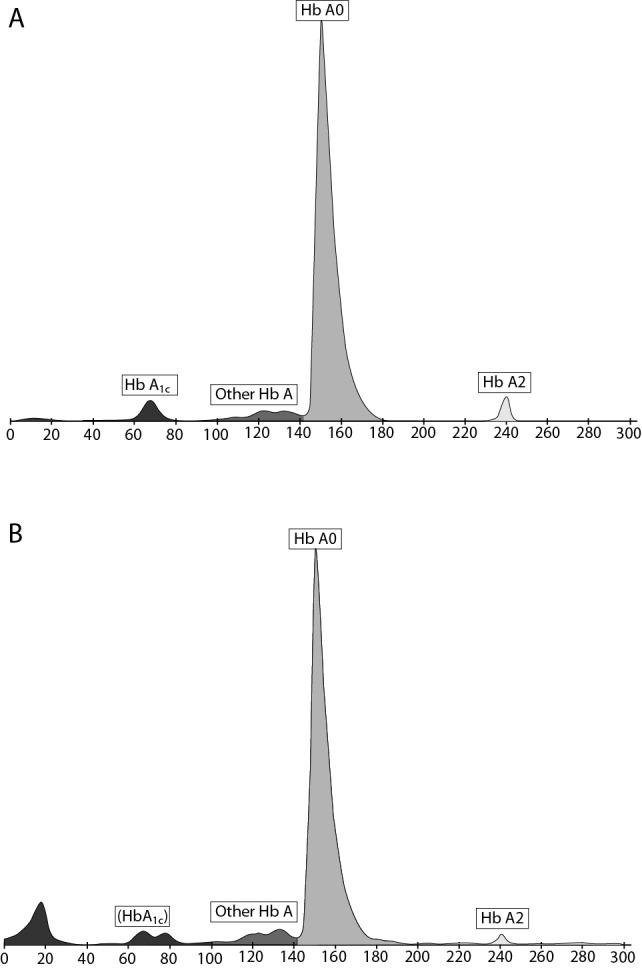
Haemoglobin A_1c_ analysis by Capillarys 2 Flex Piercing. (A) Normal control. (B) HbH samples - HbA1c cannot be measured (in parenthesis) due to overlapping with the HbBart’s fraction.

**Figure 2:**
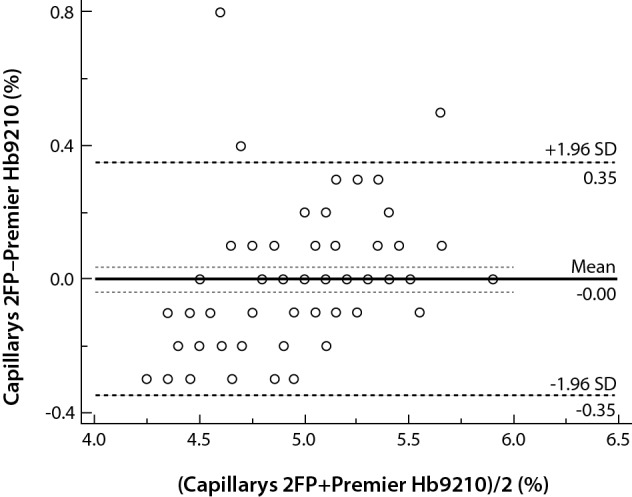
Bland-Altman plot showing the differences in haemoglobin A_1c_ results between Capillarys 2 Flex Piercing (Capillarys 2FP) and Premier Hb9210 systems in thalassaemia samples (NGSP units, %). Solid line (mean) – mean difference. Dotted lines - 95% confidence interval of mean difference. Dashed lines (± 1.96 SD) - ± 1.96 standard deviation of mean difference.

Among the 98 thalassaemia samples, HbA_1c_ was not detected by Capillarys 2FP in three samples with HbH (Figure 1). For the remaining 95 thalassaemia samples with detectable HbA_1c_, the Bland-Altman plot showed that the absolute bias of HbA_1c_ was 0.00 ± 0.35% between Capillarys 2FP and Premier Hb9210 systems, and 3.4% of values were discordant (*i.e*., differences outside the range mean ± 2SD) (Figure 2). A significant positive bias above 7% was found only in two HbH samples.

To validate these results, the samples of eight thalassemias including two α-thalassemia silent, two α-thalassemia trait, two HbH and two β-thalassemia trait, were analysed by IFCC reference method HPLC/CE for HbA_1c_. The results showed that the HbA_1c_ concentrations obtained by Capillarys 2FP were consistent with the results obtained by IFCC HPLC/CE reference method, with relative bias below ± 6% (Table 1).

**Table 1:** HbA_1c_ concentrations obtained by IFCC and Capillarys 2FP

	**Genotype**	**IFCC reference method**		**Capillarys 2FP**		**Relative bias** **(%)**
**IFCC units, mmol/mol**	**NGSP units, %**	**IFCC units, mmol/mol**	**NGSP units, %**
1	-α^3.7^/aa	39	5.7	41	5.9	3.5
2	-α^4.2^/aa	40	5.8	42	6.0	3.4
3	--^SEA^/aa	30	4.9	28	4.7	- 4.1
4	--^SEA^/aa	29	4.8	29	4.8	0.0
5	-α^3.7^/--^SEA^	21	4.1	23	4.3	4.9
6	-α^3.7^/--^SEA^	31	5.0	32	5.1	2.0
7	CD41-42	31	5.0	30	4.9	- 2.0
8	CD41-42	38	5.6	40	5.8	3.6
HbA_1c_ - haemoglobin A_1c_. IFCC - International Federation of Clinical Chemistry and Laboratory Medicine. Capillarys 2FP - Capillarys 2 Flex Piercing. NGSP - National Glycohemoglobin Standardization Programme. The relative bias was calculated in NGSP units, as the Capillarys 2FP value against the IFCC reference method value. The proficiency testing acceptance limit ± 6% of CAP was set as the accuracy limit (*25*).						

### Thalassemia screening

**Figure 3:**
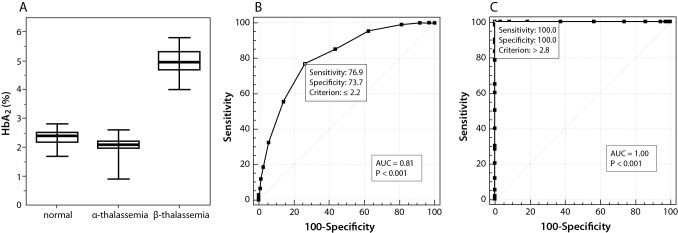
The values of haemoglobin A_2_ (HbA_2_) were analyzed by Capillarys 2 Flex Piercing for normal controls and thalassaemia. (A) The box-plots show the frequency of HbA_2_ in normal control, α-thalassaemia silent/trait and β-thalassaemia trait. The horizontal line in each box represents the median values. The upper and lower limits of each box correspond to the 25th and 75th percentile values. The highest and lowest horizontal bars represent the minimum and maximum values.  (B) For screening α-thalassaemia silent/trait, the receiver operating characteristic curve (ROC) shows an area under curve (AUC) of 0.80 (95% confidence interval, 0.78 - 0.84) and cutoff value ≤ 2.2% (sensitivity 76.9%, specificity 73.7%).  *(C) For screening β-thalassaemia trait, ROC shows AUC of 1.00 (95% confidence interval, 0.99 - 1.00) and cutoff value > 2.8% (sensitivity 100%, specificity 100%).*

The HbA_2_ concentration in α-thalassaemia silent/trait (0.9 - 2.6%) was significantly lower than normal controls (1.7 - 2.8%) (P < 0.001, Figure 3A). ROC analysis showed an area under curve (AUC) of 0.81 (95% confidence interval, 0.78 - 0.84) and the optimal cut-off value was ≤ 2.2% with a sensitivity of 76.9% and specificity of 73.7% (Figure 3B).

The HbA_2_ concentration in β-thalassaemia trait (4.0 - 5.6%) was significantly higher than normal controls (1.7 - 2.8%) (P < 0.001, Figure 3A). ROC analysis demonstrated an AUC of 1.00 (95% confidence interval, 0.99 - 1.00) and the optimal cut-off value was > 2.8% with sensitivity 100% and specificity 100% (Figure 3C).

## Discussion

Our results indicate that the presence of α-thalassaemia silent/trait and β-thalassemia trait had no significant effect on the accuracy of HbA_1c_ measurement by Capillarys 2FP. Previous studies have also revealed agreement between Capillarys 2FP and Trinity Biotech Ultra^2^ (boronate affinity chromatography method) or Adams Arkray HA-8160 (ion-exchange HPLC method) systems for β-thalassemia samples (*14*, *26*). However, HbA_1c_ concentrations in some HbH samples became immeasurable as HbH was separated as the first fraction and the HbBart’s fraction overlapped with HbA_1c_. Thus, Capillarys 2FP could provide valuable information (HbH and/or HbBart’s) for immeasurable samples. For some measurable HbH samples by Capillarys 2FP, the results were consistent with that by IFCC reference method.

The limitations of our study are the insufficient number of HbH samples and IFCC reference method confirmed samples. Another limitation of our study is the lack of β-thalassemia intermedia/major samples. The β-thalassemia is characterized by variable increases in HbF and unusually high HbA_2_ concentrations. Our previous study found that an increased proportion of HbF could affect the HbA_1c_ results using Capillarys 2FP (*5*). Thus, the β-thalassemia intermedia/major sample with highly elevated HbF (> 10%) could interfere the HbA_1c_ measurement by Capillarys 2FP. Capillarys 2FP could report an accurate HbA_1c_ value in β-thalassaemia trait and provide valuable information (elevated HbA_2_ and/or HbF) for β-thalassemia intermedia/major. Further investigation is warranted using a larger number of thalassemia intermedia/major patient samples.

HbA_2_ is well separated and quantified by the HbA_1c_ programme on Capillarys 2FP, while most other HbA_1c_ methods are not able to separate HbA_2_ from HbA_0_. In the present study, we found the optimal HbA_2_ cut-off values for screening samples with α-thalassaemia silent/trait or β-thalassemia trait were ≤ 2.2% and > 2.8%, respectively, which was consistent with the previous report for β-thalassemia (*10*). In addition, abnormal bands could also provide valuable information for thalassemia screening (HbH and/or HbBart’s for HbH disease, HbF for β-thalassemia). The HbA_2_ concentrations obtained with the HbA_1c_ programme needs to be corrected to obtain the real HbA_2_ concentrations, which warrants further investigation. Although the HbA_2_ concentration cannot be reported, laboratories must be cautious when thalassemia is suspected based on screening during HbA_1c_ measurement and communicate with clinicians to note whether the patient is anaemic. Further confirmatory analysis for thalassemia and other indicators to diagnose diabetes are recommended.

In summary, our data, although limited, demonstrated that Capillarys 2FP HbA_1c_ system could report accurate HbA_1c_ results in thalassemia silent/trait, and HbA_2_ concentrations (≤ 2.2% for α-thalassaemia silent/trait and > 2.8% for β-thalassemia trait) and abnormal bands (HbH and/or HbBart’s for HbH disease, HbF for β-thalassemia) may provide valuable information for screening.

## References

[1] Peterson KP Pavlovich JG Goldstein D Little R England J Peterson CM What is hemoglobin A1c? An analysis of glycated hemoglobins by electrospray ionization mass spectrometry. Clin Chem. 1998;44:1951–8. 9732983

[2] World Health Organization (WHO). Use of glycated haemoglobin (HbA1c) in the diagnosis of diabetes mellitus. Abbreviated Report of a WHO Consultation. Geneva, Switzerland. WHO Press, 2011. 26158184

[3] Gillett MJ International Expert Committee report on the role of the A1c assay in the diagnosis of diabetes. Diabetes Care. 2009;32:1327–34. 10.2337/dc09-9033 19502545 PMC2699715

[4] Sacks DB Arnold M Bakris GL Bruns DE Horvath AR Kirkman MS National Academy of Clinical Biochemistry; Evidence-Based Laboratory Medicine Committee of the American Association for Clinical Chemistry. Guidelines and recommendations for laboratory analysis in the diagnosis and management of diabetes mellitus. Diabetes Care. 2011;34:e61–99. 10.2337/dc11-9998 21617108 PMC3114322

[5] Wu X Chao Y Wan Z Wang Y Ma Y Ke P A comparative evaluation of the analytical performances of Capillarys 2 Flex Piercing, Tosoh HLC-723 G8, Premier Hb9210, and Roche Cobas c501 Tina-quant Gen 2 Analyzers for HbA1c determination. Biochem Med (Zagreb). 2016;26:353–64. 10.11613/BM.2016.039 27812304 PMC5082223

[6] Zhang XM Wen DM Xu SN Suo MH Chen YQ Effects of hemoglobin variants HbJ Bangkok, HbE, HbG Taipei, and HbH on analysis of glycated hemoglobin via ion-exchange high-performance liquid chromatography. J Clin Lab Anal. 2017 April 13 [cited 2017 may 5]. [Epub ahead of print] 10.1002/jcla.22214 28407371 PMC6817093

[7] Coban E Ozdogan M Timuragaoglu A Effect of iron deficiency anemia on the levels of hemoglobin A1c in nondiabetic patients. Acta Haematol. 2004;112:126–8. 10.1159/000079722 15345893

[8] Galanello R Origa R Beta-thalassemia. Orphanet J Rare Dis. 2010;5:11. 10.1186/1750-1172-5-11 20492708 PMC2893117

[9] Weatherall DJ Clegg JB Inherited haemoglobin disorders: an increasing global health problem. Bull World Health Organ. 2001;79:704–12. 11545326 PMC2566499

[10] Urrechaga E High-resolution HbA(1c) separation and hemoglobinopathy detection with capillary electrophoresis. Am J Clin Pathol. 2012;138:448–56. 10.1309/AJCPVYW9QZ9EVFXI 22912363

[11] Lee SC Wang LH Tsai SM Fang HY Tsai LY Effects of the Hb E, Hb H and Hb G-Taichung variants on HbA1c values by the Bio-Rad variant II turbo analyzer. Clin Biochem. 2011;44:1338–42. 10.1016/j.clinbiochem.2011.08.907 21871876

[12] Agilli M Yaman H Aydinl FN Hb H Interference on Measurement Of HbA1c With Ion-Exchange HPLC. Acta Inform Med. 2013;21:216–8. 10.5455/aim.2013.21.216-218 24167397 PMC3804497

[13] Pravatmuang P Sae-Ngow B Whanpuch T Leowattana W Effect of HbE and HbH on HbA1C level by ionic exchange HPLC comparing to immunoturbidimetry. Clin Chim Acta. 2001;313:171–8. 10.1016/S0009-8981(01)00670-2 11694256

[14] Ji L Yu J Zhou Y Xia Y Xu A Li W Erroneous HbA1c measurements in the presence of β-thalassemia and common Chinese hemoglobin variants. Clin Chem Lab Med. 2015;53:1451–8. 10.1515/cclm-2014-0598 25720069

[15] Al-Fadhli SM Ahmad AA Al-Jafar HA Effect of sickle cell trait and B-Thalassemia minor on determinations of HbA1c by an immunoassay method. Saudi Med J. 2001;22:686–9. 11573113

[16] Polage C Little RR Rohlfing CL Cole TG Roberts WL Effects of beta thalassemia minor on results of six glycated hemoglobin methods. Clin Chim Acta. 2004;350:123–8. 10.1016/j.cccn.2004.07.015 15530468

[17] Gallo E Pich P Ricco G Saglio G Camaschella C Mazza U The relationship between anemia, fecal stercobilinogen, erythrocyte survival, and globin synthesis in heterozygotes for beta-thalassemia. Blood. 1975;46:693–8. 1174704

[18] Mosca A Paleari R Ivaldi G Galanello R Giordano PC The role of haemoglobin A2 testing in the diagnosis of thalassaemias and related haemoglobinopathies. J Clin Pathol. 2009;62:13–7. 10.1136/jcp.2008.056945 19103851

[19] Simon M Hoover JD Effect of sample instability on glycohemoglobin (HbA1) measured by cation-exchange chromatography. Clin Chem. 1982;28:195–8. 6799223

[20] John WG Little R Sacks DB Weykamp C Lenters-Westra E Hornsby T Multicentre evaluation of the Premier Hb9210 HbA1c analyser. Clin Chem Lab Med. 2015;53:319–27. 10.1515/cclm-2014-0589 25274956 PMC5524374

[21] Lin M Wang Q Zheng L Huang Y Lin F Lin CP Prevalence and molecular characterization of abnormal hemoglobin in eastern Guangdong of southern China. Clin Genet. 2012;81:165–71. 10.1111/j.1399-0004.2011.01627.x 21231928

[22] Cai SP Wall J Kan YW Chehab FF Reverse dot blot probes for the screening of beta- thalassemia mutations in Asians and American blacks. Hum Mutat. 1994;3:59–63. 10.1002/humu.1380030110 8118466

[23] Jeppsson JO Kobold U Barr J Finke A Hoelzel W Hoshino T Approved IFCC reference method for the measurement of HbA1c in human blood. Clin Chem Lab Med. 2002;40:78–89. 10.1515/CCLM.2002.016 11916276

[24] National Glycohemoglobin Standardization Programme (NGSP). HbA1c methods: Effects of Hemoglobin Variants (HbC, HbS, HbE and HbD traits) and Elevated Fetal Hemoglobin (HbF). Available at: http://www.ngsp.org/interf.asp. Accessed April 22nd 2017.

[25] National Glycohemoglobin Standardization Programme (NGSP). College of American Pathologists (CAP) Survey Data. Available at: http://www.ngsp.org/CAPdata.asp. Accessed April 22nd 2017.

[26] Marinova M Altinier S Caldini A Passerini G Pizzagalli G Brogi M Multicenter evaluation of hemoglobin A1c assay on capillary electrophoresis. Clin Chim Acta. 2013;424:207–11. 10.1016/j.cca.2013.06.014 23792069

